# MoO_3_ Thickness, Thermal Annealing and Solvent Annealing Effects on Inverted and Direct Polymer Photovoltaic Solar Cells

**DOI:** 10.3390/ma5122521

**Published:** 2012-11-27

**Authors:** Sylvain Chambon, Lionel Derue, Michel Lahaye, Bertrand Pavageau, Lionel Hirsch, Guillaume Wantz

**Affiliations:** 1 University Bordeaux, CNRS, IMS, UMR 5218, 33400 Talence, France; E-Mails: sylvain.chambon@ims-bordeaux.fr (S.C.); lionel.derue@ims-bordeaux.fr (L.D.); lionel.hirsch@ims-bordeaux.fr (L.H.); 2 University Bordeaux, CNRS, ICMCB, UPR 9048, 33600 Pessac, France; E-Mail: lahaye@icmcb-bordeaux.cnrs.fr; 3 University Bordeaux, CNRS, RHODIA, LOF, UMR 5258, 33600 Pessac, France; E-Mail: Bertrand.pavageau@eu.rhodia.com

**Keywords:** organic solar cells, MoO_3_, diffusion processes, inverted architecture, thermal annealing, solvent annealing

## Abstract

Several parameters of the fabrication process of inverted polymer bulk heterojunction solar cells based on titanium oxide as an electron selective layer and molybdenum oxide as a hole selective layer were tested in order to achieve efficient organic photovoltaic solar cells. Thermal annealing treatment is a common process to achieve optimum morphology, but it proved to be damageable for the performance of this kind of inverted solar cells. We demonstrate using Auger analysis combined with argon etching that diffusion of species occurs from the MoO_3_/Ag top layers into the active layer upon thermal annealing. In order to achieve efficient devices, the morphology of the bulk heterojunction was then manipulated using the solvent annealing technique as an alternative to thermal annealing. The influence of the MoO_3_ thickness was studied on inverted, as well as direct, structure. It appeared that only 1 nm-thick MoO_3_ is enough to exhibit highly efficient devices (PCE = 3.8%) and that increasing the thickness up to 15 nm does not change the device performance.

## 1. Introduction

Organic photovoltaic solar cells (OSC) have been the subject of increasing attention around the world for the last 20 years as a potential source of renewable energy. OSC offer multiple advantages, such as low production costs, reduced weight, potential flexibility and power conversion efficiencies (PCE) approaching values of the amorphous silicon industry [1,2]. Since 2005, so-called inverted OSC have been developed, which exhibit improved air stability compared to conventional direct structures [3,4,5,6]. In an inverted OSC, electrons are collected by the indium tin oxide (ITO) bottom electrode through transparent electron selective layers made of materials such as calcium [7,8], titanium oxide (TiO_x_) [9,10], zinc oxide (ZnO) [11,12,13], cesium carbonate (Cs_2_CO_3_) [14,15] or aluminum-doped zinc oxide [9]. The holes are collected from the evaporated top electrode, generally across a thin film of hole selective layer, such as molybdenum oxide (MoO_3_) or poly(3,4-ethylenedioxythiophene) doped with poly(4-styrenesulfonate) (PEDOT-PSS). The top electrode is generally made of silver, since air exposure leads to the formation of silver oxide; the latter exhibits a higher work function, thus enhancing hole collection [16].

Molybdenum trioxide layer is a widely used hole selective layer in replacement of the PEDOT:PSS. It is mostly used in inverted solar cells [11,17,18,19,20], but can also be found in direct architectures [8,21,22]. It has the interesting advantage to give the devices better air stability (shelf-lifetime) in direct [8], as well as in inverted [19], architectures. This article focuses on the optimization of solar cells built with this kind of layer, both in direct and inverted structures. It involves the study of the influence of the MoO_3_-thickness, as well as the search for the optimal procedure, in order to obtain the desired nano-morphology of the active layer. Indeed, the optimal morphology of the active layer can be achieved by different ways: thermal annealing [23,24,25,26,27], solvent annealing [28], use of high boiling point solvents [29,30,31,32] and/or additives [33,34,35], all of which can have an impact on the other layers. The aim of this paper is to study the influence of two techniques, namely thermal annealing and solvent annealing, combined with the influence of the MoO_3_ thickness.

## 2. Results and Discussion

### 2.1. Influence of the Thermal Annealing on the Performances

In the organic photovoltaic field, thermal treatments of the active layers are commonly used to promote the phase segregation of the donor and acceptor materials in order to achieve the optimal morphology of the bulk heterojunction. However, this technique is versatile, as the degree of phase separation will depend on many factors, such as heating temperature and time [27] or molecular weight and regioregularity of the polymer [36,37]. Moreover, the thermal treatment can be performed before or after the top electrode deposition, which will also impact the performance of the devices [27]. In a first experiment, the influence of annealing the whole device is studied, *i.e.*, the thermal treatment is performed on a completed solar cell with the following structure: Glass/ITO/TiO_x_/P3HT:PCBM/MoO_3_/Ag. Table 1 summarizes the impact of the treatment on the performance as a function of the MoO_3_ thickness.

First of all, one can observe that in all cases, the short-circuit current increases by a factor two to three after the annealing process. This increase can be related to the well-known improvement of the charge creation and collection resulting from the phase separation of the blend.

**Table 1:** J_sc_, V_oc_, FF and PCE of devices prepared with 1 to 15 nm of MoO_3_ before and after thermal annealing at 170 °C during 10 min of the completed solar cell with the following structure: Glass/ITO/TiO_x_/P3HT:PCBM/MoO_3_/Ag.

MoO_3_ Thickness (nm)	Thermal annealing	J_sc_ (mA.cm^−2^)	V_oc_ (V)	FF	PCE (%)
**1**	Before	2.42	0.63	0.43	0.65
	After	8.48	0.54	0.41	1.91
**3**	Before	2.63	0.62	0.43	0.70
	After	8.06	0.48	0.37	1.42
**5**	Before	3.40	0.61	0.45	0.93
	After	7.05	0.51	0.45	1.60
**7**	Before	2.91	0.62	0.44	0.79
	After	7.72	0.44	0.38	1.32
**10**	Before	2.95	0.62	0.44	0.81
	After	7.94	0.52	0.44	1.81
**15**	Before	3.55	0.61	0.43	0.92
	After	7.13	0.49	0.41	1.44

However, efficiencies barely reach 1.9% in some of the cases, which is unusual for a system like P3HT:PCBM. One can observe that the V_oc_ decreases dramatically, from 0.60 V for unannealed devices to 0.44 V after thermal annealing in the worst case. This drop in V_oc_ is too important to be related only to the organization of P3HT and/or PCBM, as shown by Vandewal *et al.* [38]. Other processes might be involved during this step, such as diffusion of MoO_3_ and/or Ag in the active layer. Indeed, diffusion of MoO_3_ could create recombination centers, explaining therefore the decrease in V_oc_.

**Figure 1:**
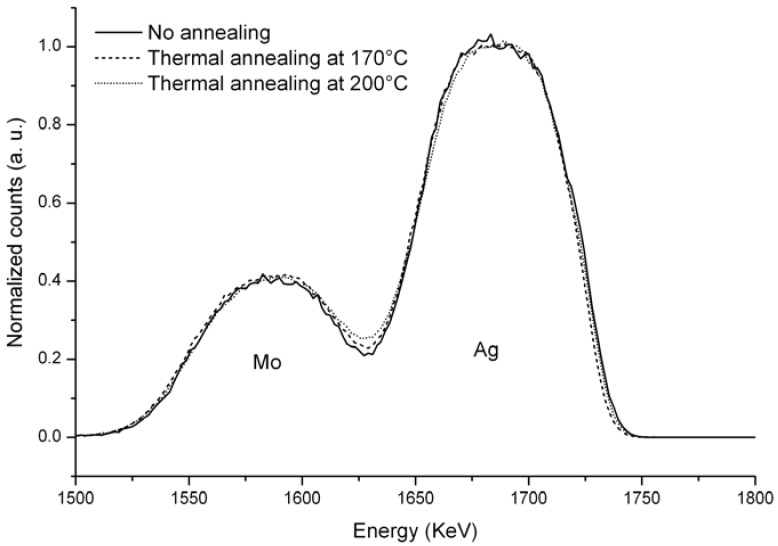
Ag and Mo signals for C/MoO_3_/Ag samples untreated (solid line), thermally treated at 170 °C (dashed line) and 200 °C (dotted line). Incident ^4^He^+^ ion energy was set at 2 MeV and an angle of detection at 160 °.

In order to detect elemental diffusion in the different layers Rutherford Backscattering Spectrometry (RBS) and Auger Electron Spectrometry (AES) were performed. RBS measurements were carried out on MoO_3_ (100 nm)/Ag (80 nm) bilayer structures deposited on glassy carbon substrates. Three kinds of samples were studied, a pristine MoO_3_/Ag sample, and two others thermally annealed for 10 min at 170 °C and 200 °C, respectively. Figure 1 displays the spectra of three Carbon/MoO_3_/Ag samples without thermal treatment, after 10 min at 170 °C and after 10 min at 200 °C. No clear differences are observed in the shape of both signals, indicating that no interdiffusion is observed with the accuracy of this technique, even if some slight differences can be detected at the Mo/Ag interface. The point where the Ag falling edge meets the Mo rising edge increases with increasing thermal treatment temperature. For thermally annealed samples, the respective Ag and Mo falling and rising slopes are slightly less pronounced than in the case of the untreated sample. These results can indicate a small interdiffusion of Ag and MoO_3_ induced by the thermal treatment. However, these slight changes might also be due to changes in the superficial and/or interfacial roughness of the layers.

**Figure 2:**
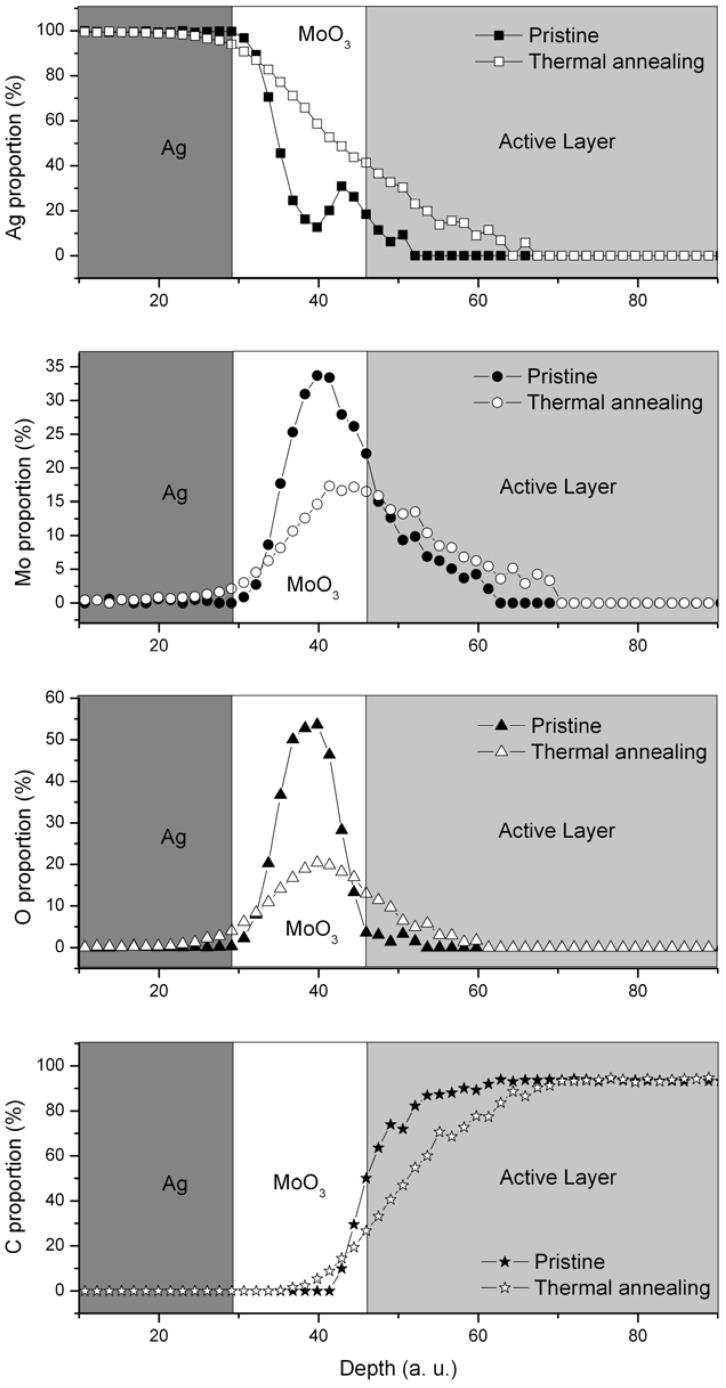
Ag (square), Mo (circle), O (triangles) and C (star) relative proportion at different depth for pristine (solid symbols) and thermal annealed (open symbols) multilayer samples (glass/ITO/TiO_x_/P3HT:PCBM/MoO_3_/Ag). The thermal annealing step was performed on the completed device. The relative proportion of each element takes also into account the contribution of the sulphur, which is not plotted. The grey zones represent an estimation of each layer location as a guideline for the reader.

Elementary depth profiles were performed on pristine and thermal annealed (170 °C for 10 min) multilayer samples (Glass/ITO/TiO_x_/P3HT:PCBM/MoO_3_/Ag) using Auger Electron Spectroscopy (AES) coupled with argon etching. The thicknesses for Ag, MoO_3_ and active layers were 80, 15 and 240 nm, respectively. Figure 2 displays the relative proportion of Ag, Mo, O and C as a function of the depth for the pristine and the thermal annealed sample.

In the case of a pristine sample, a global observation of the elements’ profiles indicates that the edges are relatively sharp, suggesting that the interfaces are well defined. If one focuses on the Ag profile, a diffusion of silver atoms in MoO_3_ and organic layers can be observed. Molybdenum seems also to migrate slightly in the active layer, while oxygen does not. These unexpected results can be explained by the technique used to create the profile. Indeed, argon etching can induce surface rugosity and/or elemental diffusion, which can explain the observed interdiffusion. Actually, such diffusion should be detected by the RBS analysis without any doubt. In case of RBS, the linear energy deposition is low compared to that of Ar etching. In conclusion, AES coupled with argon etching can only be used in this case to perform relative comparison between samples and can only give qualitative results.

As shown in Figure 2, the elements’ profiles are different in the case of thermally annealed sample. The Ag edge is not as sharp as in the pristine sample, and one can clearly observe that the silver diffuses deeply into the organic layer, passing through the molybdenum oxide layer. The molybdenum oxide layer loses also its consistency, and the migration of oxygen and molybdenum into the active layer is slightly observed. After 10 min at 170 °C, the interfaces have lost their cohesion: the MoO_3_ layer evolved into a molybdenum oxide silver alloy, and the first nanometers of the active layer present a significant amount of silver and traces of molybdenum and oxygen.

These results can be linked to the changes in the performances of the device. Indeed, thermal annealing treatment of the whole structure (glass/ITO/TiO_x_/P3HT:PCBM/MoO_3_/Ag) induces the phase segregation between the donor (P3HT) and the acceptor material (PCBM), leading to an optimal morphology for exciton dissociation and charge transport. This can be observed with the improvement of the short-circuit current (From 2–3 mA.cm^−2^ to 7–8 mA.cm^−2^). Concomitantly, species from the top electrode tend to migrate toward the organic layer, and the thin MoO_3_ layer is transformed into a silver MoO_3_ alloy. These effects can be linked to the low performances of the thermally annealed devices (Table 1). One might suggest that the presence of silver in the organic active layer can act as recombination centers and/or the resulting molybdenum-silver alloy has lost it hole extraction properties. Experiments are ongoing to further confirm these assumptions.

**Figure 3:**
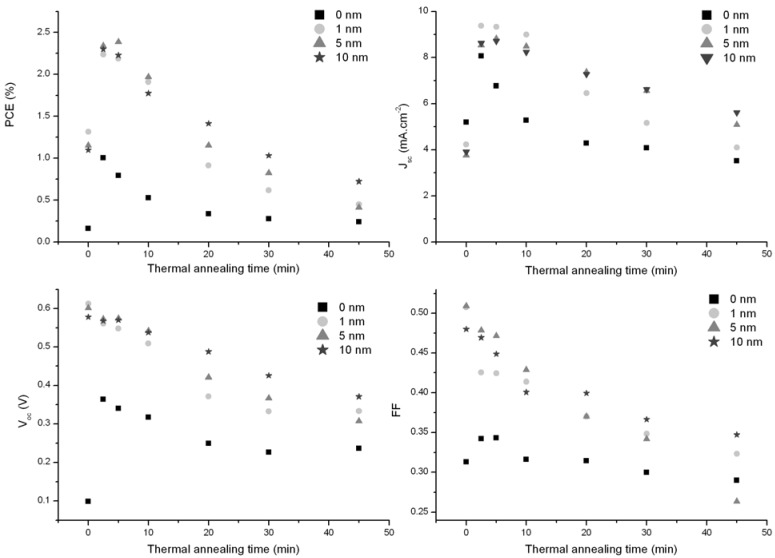
Variation of the power conversion efficiency, short-circuit current, open-circuit voltage and fill factor of devices with 0 (square), 1 (circle), 5 (triangle) and 10 nm (star) MoO_3_ thick layers as a function of the thermal annealing time.

Finding the optimal PV performances by thermal annealing treatment consists in finding the balance between promoting the phase segregation to obtain the optimal morphology and limiting the diffusion of silver into the molybdenum oxide and the active layer. Four samples, with different MoO_3_ layer thicknesses (0, 1, 5 and 10 nm), were submitted to a thermal annealing treatment at 170 °C for various durations. The variation of the PV characteristics (PCE, J_sc_, V_oc_, FF) *versus* the annealing time are plotted in Figure 3. For the three samples with MoO_3_ layer, the optimum thermal annealing time is found between 2.5 and 5 minutes. Longer thermal treatments lead to a dramatic loss of the photovoltaic properties. Devices with a very thin MoO_3_ layer lose their PV properties faster than devices with thick layers. In fact, for the device with 1 nm-thick MoO_3_ layer, a significant drop in the V_oc_ appears between 10 and 20 min of thermal treatment, while the device with 10 nm thick MoO_3_ layer does not show the same drop. As V_oc_ can be influenced by the charge extraction properties of the electrodes [39,40,41] and recombination [42,43], it can be suggested that the faster decrease of this PV parameter is due to a faster loss of coherence of the MoO_3_ layer and/or faster diffusion of silver through the oxide layer.

Thermal treatment of the whole device appears to be not suitable for inverted solar cells with molybdenum oxide as HTL and silver as top electrode. The thermal treatment was then carried out at different stages of the fabrication of the device: before and after the deposition of MoO_3_. In all the cases, the silver electrode was deposited after the thermal treatment in order to avoid its diffusion in the active layer. Results are summarized in Table 2.

When the thermal treatment is applied just after the active layer deposition, one can observe an increase of the J_sc_ up to 9 mA.cm^−2^ for devices with 10 or 15 nm-thick molybdenum oxide layers. As explained earlier, it is related to a more efficient nano-morphology. V_oc_ values fluctuate between 0.50 to 0.53 V. Although low, this value is commonly measured in our group in the case TiO_x_ and MoO_3_ based inverted devices with P3HT:PCBM with well defined active layer morphology [44]. However, the performances remain quite low, below 2%, mostly due to a very poor fill factor. Interfacial problems or unfavorable vertical segregation can be the cause of low FF. Indeed, Van Bavel *et al.* showed that thermal annealing provokes vertical phase segregation, which is favorable for direct structures, and, as a consequence, unfavorable for inverted architectures [45].

**Table 2:** J_sc_, V_oc_, FF and PCE of devices prepared with 1 to 15 nm of MoO_3_. The thermal annealing step (170 °C, 10 min) was performed before or after MoO_3_ deposition.

MoO_3_ thickness (nm)	Thermal annealing	J_sc_ (mA.cm^−2^)	V_oc_ (V)	FF	PCE (%)
**1**	Before	8.11	0.52	0.38	1.61
	After	3.56	0.28	0.37	0.38
**3**	Before	7.74	0.51	0.38	1.50
	After	4.95	0.34	0.38	0.64
**5**	Before	7.45	0.53	0.39	1.55
	After	4.91	0.28	0.45	0.54
**7**	Before	8.98	0.50	0.36	1.61
	After	4.92	0.35	0.40	0.69
**10**	Before	9.15	0.50	0.36	1.62
	After	4.46	0.34	0.39	0.58
**15**	Before	9.27	0.50	0.35	1.64
	After	4.69	0.30	0.50	0.56

When the thermal treatment occurs while the MoO_3_ layer is on top of the active layer, the performances drop significantly whatever the thickness of MoO_3_ is. J_sc_ increases slightly (up to 4–5 mA.cm^−2^), but does not reach the common values for P3HT:PCBM bulk heterojunction with optimal morphology. The low short-circuit current can be the result of charge extraction problems. Not only the J_sc_ remains low, but also the V_oc_ drops to uncommonly low values, between 0.25 and 0.35 V, which are unusual for solar cells based on P3HT and PCBM. These observations reveal that a modification at the MoO_3_/Active layer interface occurred during the thermal annealing process and led to a hole extraction impediment and/or high recombination level.

**Figure 4:**
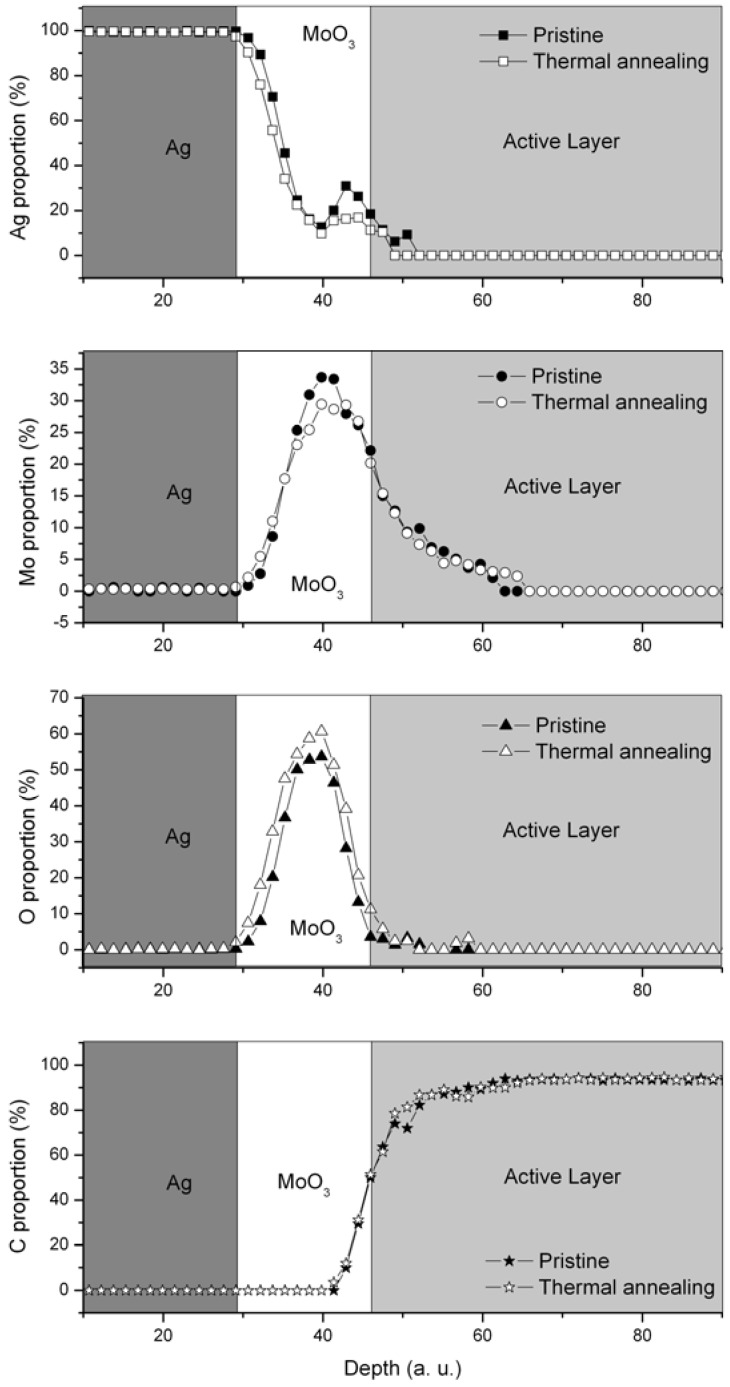
Ag (square), Mo (circle), O (triangles) and C (star) relative proportion at different depths for pristine (solid symbols) and thermally annealed (open symbols) multilayer samples (glass/ITO/TiO_x_/P3HT:PCBM/MoO_3_/Ag). The thermal annealing step was performed just before the silver electrode deposition. The relative proportion of each element takes also into account the contribution of the sulphur, which is not shown. The grey zones represent an estimation of each layer location as a guideline for the reader.

Diffusion of molybdenum oxide in the active layer could have been an explanation, as it would create recombination centers in the bulk heterojunction, therefore lowering V_oc_ and J_sc_. However the diffusion process was discarded by AES coupled with argon etching. In fact, the results (Figure 4) indicate that no diffusion of molybdenum or oxygen occurred during the thermal annealing process of the Glass/ITO/TiO_x_/P3HT:PCBM/MoO_3_ layer. The loss of cohesion of the MoO_3_ layer was also suggested to be responsible for the drop in the PV properties, but both the optical and atomic force microscopy results show no relevant differences in the film morphology and RMS roughness before and after thermal annealing (See Supplementary Information for AFM images and RMS roughness values), which discards this hypothesis. As the thermal annealing is carried out on a hot plate under a nitrogen atmosphere, one can suggest MoO_3_ is reduced, therefore modifying the position of HOMO and LUMO and leading to the loss of its holes extraction properties. It is also interesting to note that the silver top electrode slows down the dramatic loss of the PV properties. In fact, when the thermal treatment is performed on the whole device (Table 1), the V_oc_ and J_sc_ values are higher than those of devices with thermal annealing performed before silver electrode deposition (Table 2). The silver layer acts as a protecting layer for the molybdenum oxide layer and avoids its modification. The protection mechanism involved here is not fully understood and experiments are ongoing to measure the energy levels of the MoO_3_ in the different cases in order to verify the eventual formation of extraction/injection barrier.

### 2.2. Influence of Solvent Annealing on Performance

**Figure 5:**
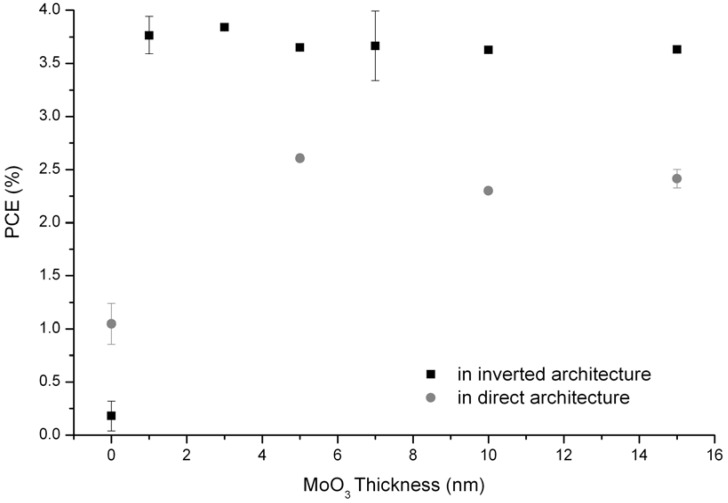
Power Conversion Efficiencies of inverted (black square) and direct (grey circle) devices with molybdenum oxide thicknesses varying from 1 to 15 nm and active layer prepared with the solvent annealing technique.

Thermal treatments appear not to be adapted for inverted solar cells based on TiO_x_ and MoO_3_ interlayers. Another approach in order to obtain efficient active layer morphology is the solvent annealing technique [28,31]. The procedure consists in slowing down the evaporation process of solvents in order to better self-organize the materials in nanodomains. Inverted and direct organic solar cells with various thicknesses of molybdenum oxide were fabricated using the solvent annealing procedure. Results are summarized in Table 3 and Figure 5.

**Table 3:** Photovoltaic characteristics of inverted devices prepared with different molybdenum oxide thicknesses and via solvent annealing technique.

	MoO_3_ thickness (nm)	J_sc_ (mA.cm^−2^)	V_oc_ (V)	FF	PCE (%)
**INVERTED**	**0**	4.56	0.12	0.29	0.18
	**1**	12.32	0.52	0.58	3.76
	**3**	12.49	0.53	0.58	3.84
	**5**	11.89	0.52	0.59	3.65
	**7**	12.50	0.53	0.55	3.66
	**10**	12.02	0.53	0.57	3.63
	**15**	12.20	0.53	0.56	3.63
**DIRECT**	**0**	8.68	0.34	0.35	1.05
	**5**	8.17	0.56	0.57	2.61
	**10**	7.32	0.57	0.56	2.30
	**15**	7.83	0.57	0.55	2.42
	**20**	7.09	0.57	0.54	2.17
	**25**	7.44	0.58	0.58	2.49

First of all, it has to be pointed out that, in inverted and direct structures, MoO_3_ is necessary for efficient devices using silver electrodes. Indeed, without the presence of the interlayer, devices present really poor performance with a very low V_oc_, 0.12 V and 0.34 V for inverted and direct devices, respectively. These results are consistent with the absence of a strong charge selective layer, in this particular case the hole selective layer. However, as soon as MoO_3_ is present, the devices present good performances.

For inverted structures, even 1 nm is enough to obtain reasonable characteristics (J_sc_ = 12.32 mA.cm^−2^, V_oc_ = 0.52 V, FF = 0.58, PCE = 3.76%). Tao *et al.* showed that devices with 1 nm-thick MoO_3_ layer present good performances when using Ag top electrode [18]. This result was further confirmed by Lee *et al.*, who proved that starting from 1 nm-thickness, the molybdenum oxide layer makes a full coverage on the surface [46]. With increasing oxide layer thickness, the performances don’t change dramatically, and the power conversion efficiencies of devices with 1 to 15 nm lie on a plateau around 3.7%. Sun *et al.* [19] also observed little variations of performance in the case of PCDTBT:PCBM devices when varying the molybdenum oxide thickness from 6 to 22 nm. Starting from 1 nm, the MoO_3_ layer is coherent enough to act as a doping layer by extracting electrons from the valence band of the organic semi-conductor, as shown by [47,48]. One could suggest that further increase of the thickness does not affect the PV performance, because MoO_3_ is sub-stoechiometric, providing its conductive properties. In direct structures, the same plateau is observed with PCE around 2.5%. Indeed, from 5 nm up to 25 nm of MoO_3_, the devices present good performances with high V_oc_ and FF. Only J_sc_ is lower than its inverted equivalent, explaining the lower PCE. This result differs from the conclusion of Shrotriya’s paper in which an optimum was found with a 5 nm-thick MoO3 layer [22], and the decrease of J_sc_ was observed with increasing MoO_3_ thickness. This result is another evidence of the conductive nature of MoO_3_, which strongly suggests that the oxide is sub-stoechiometric.

## 3. Experimental Section

The bulk heterojunction used in this study is based on a well-known couple of materials: poly(3-hexylthiophene) (P3HT) and 1-(3-methoxycarbonyl)propyl-1-phenyl[6,6]C_61_ (PCBM), which has been extensively studied in the last decade [49]. Inverted and direct solar cells have been fabricated with the structure Glass/ITO/TiO_x_/P3HT:PCBM/MoO_3_/Ag and Glass/ITO/MoO_3_/P3HT:PCBM/Al using standard procedures. 15 × 15 mm^2^ ITO-coated glass sheets (10 Ohm square, *Kintec*) are successively cleaned in acetone, ethanol and isopropanol in an ultrasonic bath and exposed to UV-ozone for 20 min. Titanium oxide is prepared as described by Chambon *et al.* [44]. The layer thickness was measured to be 15 +/− 5 nm using a *Tencor IQ* profilometer. MoO_3_ was deposited by thermal evaporation under a secondary vacuum (10^−6^ mbar) at the rate of 0.1 nm.s^−1^. P3HT (*Plexcore OS2100*) and PCBM (99.5%) were supplied from *Plextronics* and *Solaris-Chem Inc*., respectively, and used as received. Solutions were prepared in o-dichlorobenzene, at a 1:1 weight ratio and a concentration of 20 mg/mL. Solutions were first stirred at 90 °C for 10 min and, subsequently, at 50 °C for 24 h. For samples submitted to thermal annealing, P3HT:PCBM layers were spin coated at 1000 rpm during 80 s. The thermal annealing is performed at 170 °C for 10 min on a temperature-controlled hot plate. For samples submitted to solvent annealing, the spin-coating time was reduced to 45 s. Instantly, after spin-coating, the substrates were individually placed in small closed petri dishes at room temperature for at least 2 hours. Solvent annealing is a well-known technique to promote the phase segregation of bulk-heterojunction materials by keeping the layers under a saturated solvent atmosphere [28]. The resulting P3HT:PCBM thickness was 240 +/− 10 nm. For inverted architecture, MoO_3_ (*Serac*) followed by 100 nm-thick silver electrodes were successively thermally-evaporated (MoO_3_ deposition rate: 0.1 nm.s^−1^; Ag deposition rate 0.2–0.3 nm.s^−1^) under a secondary vacuum (10^−6^ mbar) onto P3HT:PCBM through a shadow mask to define a 8.6 mm^2^ active area. For direct architecture, a 100 nm-thick aluminum top electrode was evaporated under the same conditions (Al deposition rate 0.5 nm.s^−1^). Experiments were repeated on eight individual cells to evaluate the standard deviation. The devices were characterized using a *K.H.S. SolarCelltest-575* solar simulator with AM1.5G filters set at 100 mW/cm^2^ with a calibrated radiometer (IL 1400BL). Labview controlled *Keithley 2400* SMU enabled the measurement of current density-voltage (J-V) curves every 4 seconds to observe any eventual curve modification during light exposure. Devices were fabricated and characterized under nitrogen in a set of gloveboxes (O_2_ and H_2_O < 0.1 ppm).

Rutherford Backscattering Spectroscopy was performed using the AIFIRA facility of the Centre d’Etude Nucléaire de Bordeaux Gradignan (CENBG). A 2 MeV ^4^He^+^ beam has been used. The size of the beam diameter has been set at 3mm in order to prevent degradation on the samples, and current has been set at 1 nA. The angle of detection was 160°.

Auger Electron Spectroscopy (AES, VG Microlab 310F) was used in order to carry out a depth chemical composition analysis of the multilayer samples, especially on the Ag/MoO_3_ and MoO_3_/Active layer interfaces. The AES analyses were made with an acceleration voltage of 10 keV and a beam current of 4 nA. The energy ranges investigated in this technique were the following for each element: 351 eV (AgMN1), 186 eV (MoMN3), 508 eV (OKL1), 152 eV (SLM1) and 272 eV (CKL1). The peaks in the energy spectrum were correlated to an AES database to estimate the atomic concentration. To perform the depth profile, the conditions have been optimized to ensure the best resolution in depth: low energy of the argon ions (1.5 kv), rotation of the sample under sputtering. With a beam of 400 nA and a scanning of 2 × 2 mm, the etching rate is around 0.05 nm/s.

## 4. Conclusions

In this article, it was shown that inverted polymer solar cell architectures based on TiO_x_ as electron selective and MoO_3_/Ag as hole selective layers are extremely sensitive to thermal annealing. If the whole multilayered structure is submitted to thermal treatment (170 °C), diffusion processes occur concomitantly with the beneficial reorganization of the bulk heterojunction. This process leads to the transformation of the molybdenum oxide layer into a molybdenum oxide silver alloy. Moreover, traces of silver and molybdenum were found in the first nanometers of the active layer acting as recombination centers. An optimum thermal annealing time was found between 2.5 and 5 min, which gives an optimized active layer morphology and limited silver and molybdenum diffusion. Carrying out the thermal annealing process during the other stages of fabrication of the devices (after the active layer deposition or after the MoO_3_ deposition) does not lead to high performances. The worst performances were found when the thermal annealing is carried out after MoO_3_ deposition. Modification of the energy levels of the MoO_3_ layer is suspected to be responsible for the poor performances. As the thermal annealing technique appeared not to be suitable for achieving optimal performances for devices based on MoO_3_/Ag top electrode, the solvent annealing technique was chosen as an alternative. The influence of the MoO_3_ thickness was studied and it appears that only 1 nm is enough to have high performance devices (PCE = 3.8%) and that increasing the thickness up to 15 nm does not affect the device performances.

## Supplementary Materials

Supplementary File 1
