# Supplementary material for: MoO3 Thickness, Thermal Annealing and Solvent Annealing Effects on Inverted and Direct Polymer Photovoltaic Solar Cells
Source: Materials (Basel). 2012 Nov 27;5(12):2521–36. doi: 10.3390/ma5122521 (PMC5449054; doi:10.3390/ma5122521)
Supplement: Supplementary File 1 [file materials-05-02521-s001.pdf]

Supplementary Information

AFM

AFM images of Glass/ITO/TiO_x/P3HT:PCBM and Glass/ITO/TiO_x/P3HT:PCBM/MoO_3 before and after thermal annealing (170°C – 10 min) are shown on Figure S1. The MoO_3 layer is 15 nm-thick and the RMS roughness was evaluated on a 2 μm × 2 μm area.

The RMS roughness of Glass/ITO/TiO_x/P3HT:PCBM is 1.4 and 2.0 nm respectively before (S1-A) and after (S1-C) annealing. For Glass/ITO/TiO_x/P3HT:PCBM/MoO_3 samples, the RMS roughness is 1.7 and 0.7 nm respectively before (S1-B) and after (S1-D) thermal annealing.

Figure S1. AFM height images of Glass/ITO/TiO_x/P3HT:PCBM (**A**, **C**) and Glass/ITO/TiO_x/P3HT:PCBM/MoO_3 (**B**,**D**) before (**A**, **B**) and after (**C**, **D**) thermal annealing at 170°C for 10 min.

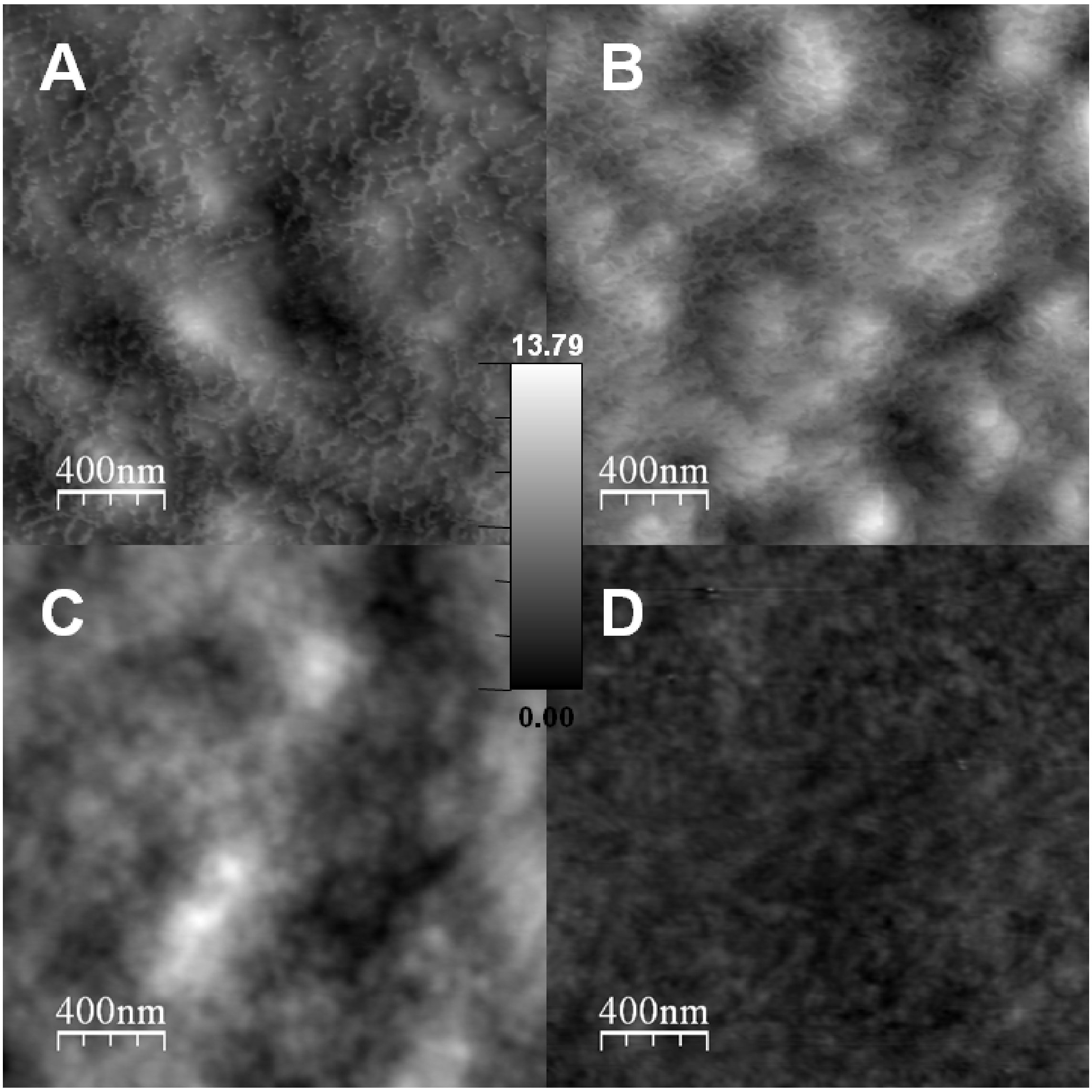

© 2012 by the authors; licensee MDPI, Basel, Switzerland. This article is an open access article distributed under the terms and conditions of the Creative Commons Attribution license (http://creativecommons.org/licenses/by/3.0/).
